# Recurrent Strokes in a Woman with a History of Thrombotic Thrombocytopenic Purpura

**DOI:** 10.1155/2023/7070189

**Published:** 2023-08-02

**Authors:** Ijele Adimora, Bingnan Zhang, Modupe Idowu

**Affiliations:** ^1^Department of Internal Medicine, The University of Texas Health Science Center at Houston, 6431 Fannin St., Houston, TX 77030, USA; ^2^Division of Cancer Medicine, The University of Texas MD Anderson Cancer Center, 1515 Holcombe Blvd., Houston, TX 77030, USA; ^3^Division of Hematology, Department of Internal Medicine, McGovern Medical School, The University of Texas Health Science Center at Houston, 6431 Fannin St., Houston, TX 77030, USA

## Abstract

Thrombotic thrombocytopenic purpura (TTP) is a microangiopathy characterized by mechanical hemolytic anemia, resulting in end-organ damage. We describe a case of TTP which presented as an ischemic stroke. The patient presented with stroke as the primary manifestation of TTP despite a normal platelet count and mildly elevated lactate dehydrogenase level (LDH). The patient underwent two transfusions of fresh frozen plasma (FFP), and ADAMTS13 levels confirmed the diagnosis of TTP after discharge. This case demonstrates the importance of maintaining a high index of suspicion for TTP in the setting of normal laboratory values and reveals the many atypical manifestations of TTP.

## 1. Introduction

Thrombotic thrombocytopenic purpura (TTP) is a microangiopathy characterized by a mechanical hemolytic anemia and a consumptive thrombocytopenia, resulting in end-organ damage from thrombotic occlusion of small vessels. TTP can either be acquired or congenital. The acquired form is autoimmune, caused by the development of antibodies to ADAMTS13, a proteolytic enzyme which cleaves von Willebrand Factor (vWF). The congenital form is rare and caused by a genetic mutation in the ADAMTS13 gene, leading to a deficiency of the corresponding protein. ADAMTS13 activity levels less than 10% in the presence of an inhibitor are diagnostic of acquired TTP. Microthrombi resulting from conglomerates of uncleaved vWF multimers and platelets can lead to renal failure, myocardial infarction, and cerebrovascular ischemia [1]. TTP left untreated has a mortality rate approximating 90%, and therefore patients with suspected TTP are treated with plasma exchange (PLEX) prior to resulting ADAMTS13 levels [2].

Hereditary TTP is caused by biallelic ADAMTS13 mutations. It typically presents in infancy but can also manifest in early adulthood. Patients who present with TTP in early adulthood may have a discernible trigger for an initial acute episode such as infection or pregnancy. Relapses are common in both the acquired and congenital forms and may not present in the same manner as the inciting clinical event [3]. While TTP should be suspected in all patients with otherwise unexplained microangiopathic hemolytic anemia and thrombocytopenia, these laboratory findings may not always precede symptoms and signs of end-organ damage, and in many cases may not be present at all. While few patients present with the classic pentad of hemolytic anemia, thrombocytopenia, neurologic deficits, renal failure, and fever, TTP in the setting of a normal to near-normal platelet is a known manifestation of hereditary TTP.

## 2. Case Presentation

The patient is a 53-year-old female with a past medical history of thrombotic thrombocytopenic purpura (TTP), asplenia, hypertension, hepatitis C, a patent foramen ovale with closure, and multiple strokes presented to our institution with a three-day history of dysequilibrium and nausea.

The patient was initially diagnosed with TTP at age 19 during admission for prolonged uterine bleeding after her first pregnancy. It is unclear how the first diagnosis was made. While splenectomy can be performed for patients with recurrent TTP, this patient underwent splenectomy after her first episode of TTP for unclear reasons. She had a recurrent episode of TTP during her second pregnancy at age 25. More recently, she had an episode of TTP after a cholecystectomy at age 48, with platelets dropping to 16,000/uL, and was treated with total plasma exchange and steroids.

Regarding her history of multiple strokes, she suffered her first stroke to the left middle cerebral artery in 2020 and was started on aspirin and statin. At this time, she was found to have a patent foramen ovale (PFO) on TEE which was thought to be contributing to the stroke; therefore, she underwent surgery for PFO closure. She remained compliant on the medical therapy and yet suffered a repeat stroke to the right corona radiata seven months later. Afterwards, she was switched from aspirin to clopidogrel and remained on a statin for stroke prevention. Despite being on optimal medical therapy, the patient suffered a third stroke to the left cerebellum just one month later. Clopidogrel was then discontinued, and she was started on apixaban 5 mg twice daily. She had been adherent to apixaban when she presented to our institution.

On presentation, the patient was well-appearing and in stable condition. She was found to have an elevated systolic blood pressure of 160 mmHg with otherwise stable vital signs. Neurologic examination was significant for subcortical slowing and aphasia from prior strokes and a new left upper extremity ataxia. The patient's complete blood count was within normal limits (white blood cell count 8.7 × 10^3^/*μ*L, hemoglobin of 12 g/dL, and platelets of 216,000/uL). A basic metabolic panel was within normal limits. A head CT without contrast displayed no evidence of an acute intracranial abnormality. Magnetic resonance imaging revealed new ischemic changes in the left anterior cerebral artery territory involving the frontoparietal paramedian cortices, confirming the diagnosis of a new stroke.

Hematology was consulted on hospital day 2. The patient was found to have a normal platelet count of 208,000/uL, elevated LDH of 281 unit/L (normal 98–192), and haptoglobin of 159 mg/dL (Table 1). The patient's pretest probability of TTP was calculated using the PLASMIC scoring system and was found to place her at a low risk of TTP. However, given her history of TTP, an ADAMTS13 activity level, a peripheral smear, and repeat hemolysis labs were obtained. Peripheral smears revealed several acanthocytes and occasional target cells with <1 schistocytes per high-power field which did not support a diagnosis of TTP. The patient continued on antiplatelet and anticoagulation therapy. The patient's platelets remained within normal range during admission, downtrending to a nadir of 195,000/uL on hospital day 3 (see Table 1). Despite resolution of symptoms and insufficient data to recommend plasma exchange, we recommend transfusion of 2 units of fresh frozen plasma (FFP) to prevent further recurrence of stroke given the high suspicion of TTP. On hospital day 4, the patient's platelets rose to 237,000/uL, and she was discharged from the primary neurology service with strict outpatient hematology and neurology follow-up with ADAMTS13 levels pending. The patient was counseled about the likely need for repeat ADAMTS13 replacement via scheduled FFP transfusions. ADAMTS13 activity level resulted after discharge at <3% with the presence of inhibitor at a titer of 1.3 BEU. The patient was eventually able to be seen in the hematology clinic several months later for assessment of prophylactic FFP infusions. ADAMTS13 levels were tested at subsequent visits and had normalized to 22%. The patient was genetically tested for congenital TTP and was found to be negative for any genetic variants.

## 3. Discussion

While there are more than 200 pathologic genetic variants of the ADAMTS13 mutation, cases of hereditary TTP make up less than 5% of all TTP cases. Many times, these genetic variants can be under-tested, leading to a delay in diagnosis. It is important, then, to use clinical history to aid in the diagnosis of hereditary TTP. Stroke occurs in 25–31% of patients with hereditary TTP, with the median age being just 19 years [4]. While stroke is not an uncommon event in patients suffering from TTP relapse, the majority of these ischemic events occur simultaneously with a drop in platelet number or other indication of hemolytic anemia.

Our patient presented with a recurrent stroke and had normal platelets, mildly elevated LDH, and normal haptoglobin, making this an atypical presentation of TTP. While her history was initially strongly suggestive of hereditary TTP, the presence of an inhibitor points to the fact that this is an atypical presentation of the acquired form. Furthermore, her unremarkable testing for genetic variants confirms this diagnosis. It is likely that the three strokes she suffered prior to this presentation were also manifestations of her acquired TTP. Treatment of hereditary TTP involves prophylactic plasma infusions, which can help prevent future end-organ ischemia. Despite our patient having the acquired form of TTP, her ADAMTS13 levels still responded to the prophylactic infusions of plasma.

Because of the high mortality associated with untreated TTP, particular attention should be paid to young or middle-aged individuals who present with recurrent strokes in the absence of obvious cardiovascular risk factors, even in the setting of normal platelet counts [5]. The development of thrombosis may precede the development of laboratory abnormalities in patients with either congenital or acquired TTP. There are rare reports of atypical TTP presenting initially as either pulmonary embolism or myocardial infarction prior to the development of marked thrombocytopenia or hemolysis [6], and therefore a high index of suspicion must be maintained even in the absence of a high pretest probability, as in our patient. Demonstrating decreased ADAMTS13 activity is a highly specific way to confirm the diagnosis of TTP [7], and it can play a role in timely therapy with either total plasma exchange or administration of FFP [8, 9]. Furthermore, caplacizumab, an anti-von Willebrand factor immunoglobulin fragment, has been shown to normalize platelet counts and prevent recurrence of TTP when used during and 30 days after administration of plasma exchange therapy [10]. The current data suggest that caplacizumab is most efficacious when used at the time of diagnosis, and there is a paucity of data regarding its use as solely a prophylactic medication [11]. It also appears that the effects of caplacizumab after discontinuation may not be long-lasting, as recurrence rates for TTP were shown to be higher during the patient follow-up period after drug cessation [12]. In addition, its high cost burden makes it even more difficult to justify its use in our patient, for whom a clinically significant benefit may not be realizable. At follow-up, the patient's ADAMTS13 levels had normalized without any further administration of plasma or immunosuppressant therapy. Because of this, the patient was managed with a watchful waiting approach and has ADAMTS13 levels checked routinely at 3-4 month intervals to guide further treatment approaches.

Our patient's case reveals the variation in presentation of TTP, the complexity of diagnosis, and demonstrates a lack of evidence that can be used to guide management in patients with recurrent episodes of TTP but normal markers of hemolysis. Further studies need to be performed in order to delineate whether prophylactic plasma transfusions, immunosuppression, or caplacizumab can be useful for recurrent episodes of autoimmune acquired TTP and how frequently these patients should be monitored.

## Figures and Tables

**Table 1:** Summary of lab values for patient on admission at the time of evaluation and at discharge.

Lab markers	On admission	At consultation	At discharge
LDH (u/L)	—	281	—
Platelet count (K/*μ*L)	217	208	195
ADAMTS13 activity (%)	—	—	3
Haptoglobin (mg/dL)	159	—	—

## Data Availability

Justifications for the empiric treatment of our patient and for the conclusions represented in our discussion are made available in the cited medical literature.
